# Behavioral Pain Assessment Implementation in Long-Term Care Using a Tablet App: Case Series and Quasi-Experimental Design

**DOI:** 10.2196/17108

**Published:** 2020-04-22

**Authors:** Mahnoor Zahid, Natasha L Gallant, Thomas Hadjistavropoulos, Eleni Stroulia

**Affiliations:** 1 Department of Psychology and Centre on Aging and Health University of Regina Regina, SK Canada; 2 Department of Computing Science University of Alberta Edmonton, AB Canada

**Keywords:** pain measurement, long-term care, nursing, technology Alzheimer disease, mHealth

## Abstract

**Background:**

Pain is often underassessed and undertreated among long-term care (LTC) residents living with dementia. When used regularly, the Pain Assessment Checklist for Seniors With Limited Ability to Communicate (PACSLAC) scales have been shown to have beneficial effects on pain assessment and management practices and stress and burnout levels in frontline staff in LTC facilities. Such scales, however, are not utilized as often as recommended, which is likely to be related to additional record-keeping and tracking over time involved with their paper-and-pencil administration.

**Objective:**

Using implementation science principles, we assessed the introduction of the PACSLAC-II scale by comparing two methods of administration—a newly developed tablet app version and the original paper-and-pencil version—with respect to the frequency of pain assessment and facility staff feedback.

**Methods:**

Using a case series approach, we tracked pain-related quality indicators at baseline, implementation, and follow-up periods. A quasi-experimental design was used to evaluate the effect of the method of administration (ie, *paper-and-pencil only* [n=18], *tablet only* [n=12], *paper-and-pencil followed by tablet app* [n=31], and *tablet app followed by paper-and-pencil* [n=31]) on pain assessment frequency and frontline staff stress and burnout levels. Finally, semistructured interviews were conducted with frontline staff to obtain perspectives on each method of administration.

**Results:**

The implementation effort resulted in a great increase in pain assessment frequency across 7 independent LTC units, although these increases were not maintained during the follow-up period. Frontline staff reported lower levels of workload in the *paper-and-pencil followed by tablet app condition* than those in the *paper-and-pencil only* (*P*<.001) and *tablet app followed by paper-and-pencil* (*P*<.001) conditions. Frontline staff also reported lower levels of workload in the *tablet-only* condition than those in the *paper-and-pencil only* condition (*P*=.05). Similarly, lower levels of emotional exhaustion were reported by frontline staff in the *paper-and-pencil followed by tablet app* condition than those in the *paper-and-pencil only* (*P*=.002) and *tablet app followed by paper-and-pencil* (*P*=.002) conditions. Finally, frontline staff reported higher levels of depersonalization in the *paper-and-pencil only* condition than those in the *tablet app only* (*P*=.008), *paper-and-pencil followed by tablet app* (*P*<.001), and *tablet app followed by paper-and-pencil* (*P*<.001) conditions. Furthermore, narrative data from individual interviews with frontline staff revealed a preference for the tablet app over the paper-and-pencil method of administration.

**Conclusions:**

This study provides support for the use of either the tablet app or the paper-and-pencil version of the PACSLAC-II to improve pain-related quality indicators, but a reported preference for and lower levels of stress and burnout with the use of the tablet app method of administration suggests that the use of the tablet app may have more advantages compared with the paper-and-pencil method of administration.

## Introduction

**Pain in Long-Term Care**

Pain is very prevalent among older adults, and its prevalence is expected to increase as Canada’s population continues to age [1]. Among older adults residing in long-term care (LTC) facilities, and especially among residents with cognitive impairments, pain is often underassessed and undertreated [2,3]. Although pain levels do not differ among older adults with and without cognitive impairment, those with cognitive impairments are less likely to report pain compared with their cognitively intact counterparts [4-6]. As a result, higher rates of pain persist among older adults with cognitive impairment because pain is not as readily addressed as it is for their cognitively intact counterparts.

Importantly, underassessed pain can have dire consequences for this population. Among residents with dementia, for example, pain can result in behavioral disturbances and, if the pain is not assessed properly, such disturbances may be misattributed to a psychiatric condition [7]. Thus, residents with dementia experiencing pain are frequently treated with psychotropic medications, such as benzodiazepines, rather than analgesic medication [8]. The increased use of benzodiazepines to treat behavioral disturbances because of undermanaged pain in this population can result in negative consequences such as an increased risk of falls [9].

Self-report pain assessments are typically considered a valid means for evaluating the subjective nature of pain [10,11]. However, as previously mentioned, assessment is more complicated when assessing pain in individuals with moderate-to-severe dementia who are often unable to accurately self-report their pain. Observational behavioral pain assessment checklists are, therefore, important tools that are used in such cases [11]. The Pain Assessment Checklist for Seniors With Limited Ability to Communicate (PACSLAC) scales are examples of evidence-based behavioral pain assessment checklists that can be used for residents with dementia [4,12]. The clinical usefulness of these scales is strongly supported by the research literature. For example, the PACSLAC, when used regularly, has been shown to improve the use of analgesic medication and, consequently, can lead to reduced pain levels observed by staff [13]. Frontline staff using the PACSLAC scales also reported reduced stress and burnout levels compared with those who did not use the PACSLAC. Finally, regular use of PACSLAC scales reduced the use of benzodiazepines, which could help address the problem of polypharmacy in LTC [11].

Although observational pain assessment tools such as the PACSLAC scales have been implemented in LTC facilities, they are not used as frequently as recommended (eg, so that each resident is assessed for pain using a standardized tool a minimum of once per week) [14]. Regular use of these tools can be hampered because of a variety of factors, including limited time, staffing issues, and extra workload associated with paper-and-pencil administration of the tool. The paper-and-pencil version of the PACSLAC-II, for example, involves manual addition of checklist scores. It is the nurses’ responsibility to diarize pain scores over time for pain-related pattern identification because PACSLAC-II scores should always be considered in relation to previous scores [15]. In addition, tools such as the PACSLAC-II should be administered a minimum of once per week for each non-self-reporting resident and, if moderate-to-severe pain is identified, it should be administered once again within 24 hours, after treatment has been implemented [14]. These guidelines require frontline staff to complete a considerable amount of paperwork requiring proper charting.

### Aims and Objectives

This study was aimed at comparing a newly developed tablet app version of the PACSLAC-II with the original paper-and-pencil version. All the potential complexities associated with the paper-and-pencil version of the PACSLAC-II, as noted above, were addressed in the development of the tablet app method of administration. The tablet app, for example, can automatically add up the scores for each administration. The tablet app can also graph pain assessment scores over time. Finally, the tablet app compiles all the pain assessments and reassessments into a PDF containing the time and date of administration, the name of the assessor, the total score on the PACSLAC-II, and all the items from the PACSLAC-II that were endorsed to result in that total score. This method of administration allows for easier charting and record-keeping.

The tablet app version of the PACSLAC-II would be considered a type of mobile health (mHealth). Although evidence for the efficacy of mHealth is still mixed, a recent meta-analysis demonstrated that mHealth interventions are more effective than comparable non-mHealth interventions in improving health outcomes with a small overall weighted effect size [16]. Moreover, mHealth has been shown to lead to improvements in symptoms, hospitalizations, and death for a variety of health conditions [17]. The use of mHealth to assist in the management of pain [18-21] and the provision of care for aging populations [22-24] is supported by recent literature. Thus, given the recent findings regarding mHealth, the development and evaluation of the tablet version of the PACSLAC-II are warranted.

The first objective of this study was to evaluate whether pain assessment frequency improved with the use of the tablet app compared with that for the paper-and-pencil method of administration of the PACSLAC-II. For this primary objective, a case series design—that is, a descriptive approach that follows groups exposed to the same intervention over a specified period [25]—was employed. Each of the 7 independent LTC units was therefore separately evaluated using this design. Using a quasi-experimental design, the second objective was to evaluate the impact of each method of administration of the PACSLAC-II on frontline staff stress and burnout levels with the use of self-report questionnaires. The third objective was to obtain the perspectives of frontline staff on each method of administration using semistructured individual interviews.

## Methods

### Participants

Participants who completed self-report questionnaires included nurses and care aides working at participating LTC units who were fluent in both written and verbal English. The original sample included 121 frontline staff with females comprising 91.7% (111/121) of the sample between the ages of 21 and 70 years (mean 42.21, SD 10.85). The staff reported up to 36 years of experience working in LTC (mean 10.31, SD 8.47) and comprised 27.3% (33/121) of nurses and 72.7% (88/121) of special care aides. Because of limitations regarding the availability of frontline staff during their shifts, only 92 of these participants completed the stress and burnout questionnaires following the implementation period. A total of 43 participants completed interviews that were used in our qualitative analysis. All participants provided informed consent before participating in this study. An additional consent form was used for the audio recording of individual interviews. Participants were provided with a Can $10 (US $7.54) gift card after completing a set of questionnaires. In addition, participants who completed an individual interview (n=43) were compensated with an additional Can $10 (US $7.54) gift card. Personal information and individual responses collected as part of the study were kept confidential. Approval for this study was granted by our institutional ethics review board. Quality indicators comprised anonymized data about pain assessment frequency (and timeliness on related intervention) from each participating LTC unit (see *Setting* section for more details).

### Setting

A total of 7 independent LTC units took part in this study. Units A through E were located in a mid-sized metropolitan area. Units A through D belonged to the same facility, but they were treated as independent units because no overlap between the frontline staff was established. Unit E was a separate LTC facility. Units F and G were separate LTC facilities located in a rural area near the mid-sized metropolitan area. Units consisted of 32, 28, 39, 40, 90, 30, and 45 beds, respectively.

### Materials

#### Tablet App Version of the Pain Assessment Checklist for Seniors With Limited Ability to Communicate-II

For this study, we used Samsung Galaxy Tab A 7.0 devices with a 7-inch display screen and a weight of 283 g. The tablet app version of the PACSLAC-II was downloaded onto each of these tablets. A document providing instructions on how to use the PACSLAC-II app on the tablet was distributed to the staff of the participating LTC units. The tablet app version of the PACSLAC-II was meant to be a simple and literal interpretation of the paper-and-pencil version of the pain assessment tool. After signing in, the staff was presented with the same set of items in the same linear order and under the same headings as the paper-and-pencil version. Patient names and demographic identifiers were not entered into the app to protect confidentiality. Data collected from the app were encrypted through https to be transmitted and stored in a back-end repository that was secured behind a firewall.

**Figure 1:**
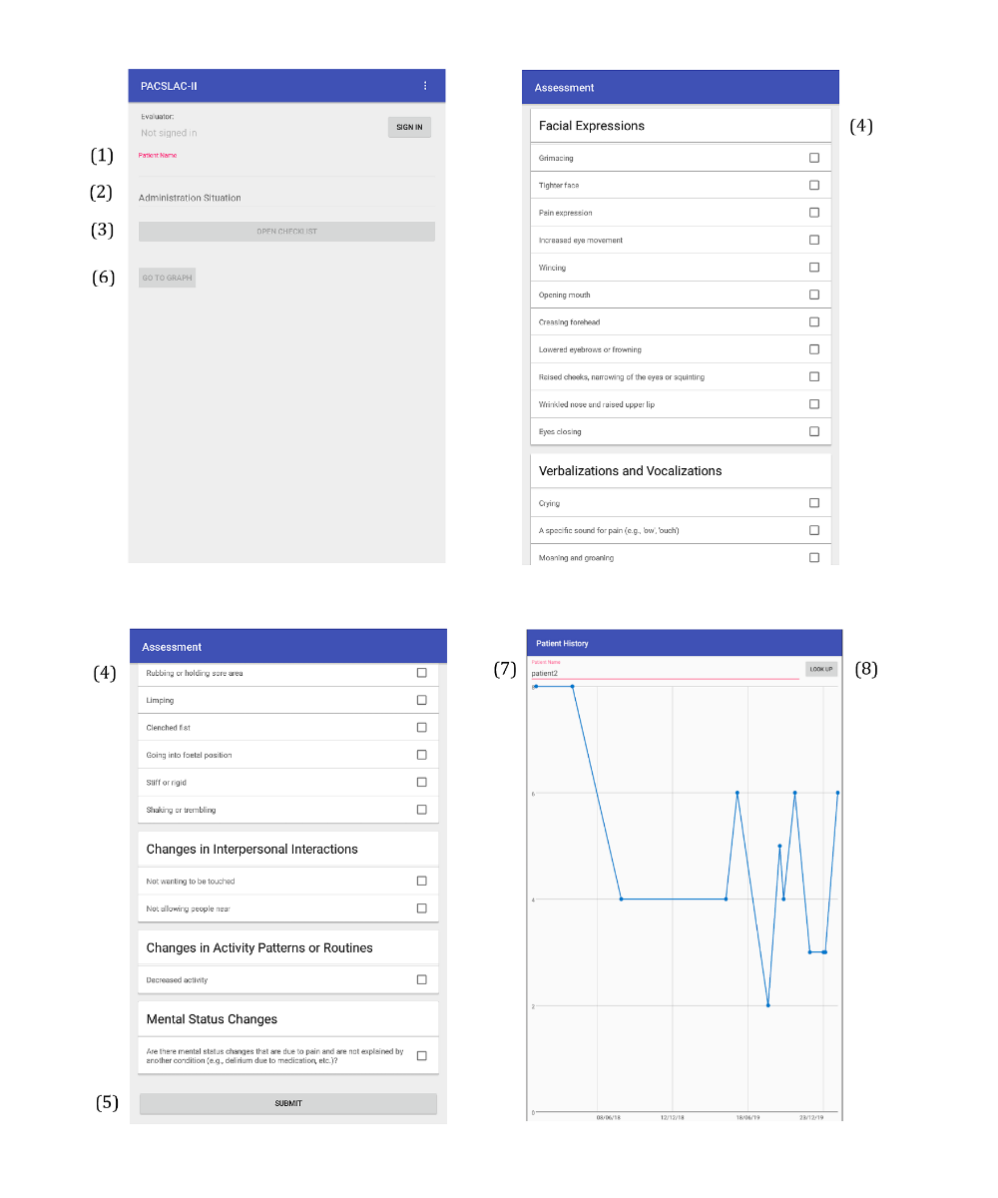
Screenshots of the Pain Assessment Checklist for Seniors With Limited Ability to Communicate (PACSLAC)-II tablet app with numbers corresponding with the instructions provided to participants. The screenshot on the bottom right of the figure shows the graph for a sample participant with the date of the PACSLAC-II administration on the horizontal axis and the PACSLAC-II score on the vertical axis.

To use the PACSLAC-II app, frontline staff would click on the PACSLAC-II icon in the apps folder on the tablet device’s home page. Once the PACSLAC-II app was open, staff would sign in using a username and password that they had created for themselves. To complete a PACSLAC-II assessment (see Figure 1 for corresponding screenshots), staff would complete the following steps: (1) enter the resident’s identification number and initials in the *patient name* box, (2) enter the location of administration in the *administration situation* box, (3) proceed to the PACSLAC-II observational checklist by clicking the *open checklist* button, (4) indicate the items by clicking the box adjacent to the applicable items for that resident, (5) click the *submit* button at the bottom of the PACSLAC-II observational checklist and to access the graph showing PACSLAC-II results over time, staff would (6) click the *go to graph* button on the bottom left of the screen, (7) enter the resident’s identification number and initials in the *look up* box, and (8) click the *patient PDF* button.

#### Web-Based Training Program

In collaboration with an instructional designer and a Web developer, an interactive and dynamic Web-based training program pertaining to pain assessment in LTC with a focus on the use of the PACSLAC-II for persons with dementia was developed. The Web-based training program included 6 core modules that were each designed to be completed within 10 to 15 min. An additional optional module was created to provide staff with the opportunity to practice using the PACSLAC-II. The Web-based training program has been deemed to be useful by nursing staff and to increase the frequency of pain assessments in LTC when the paper-and-pencil method of administration was used [26].

### Measures

#### Quality Indicators

Quality indicators measured each LTC unit’s performance with regard to pain assessment frequency and follow-up for moderate-to-severe pain. These quality indicators were adapted from a consensus protocol for pain assessment and management developed following consultation with a group of public policy and geriatric pain experts [27]. This consensus protocol has been successfully implemented in LTC facilities [2]. The protocol incorporated the following quality indicators: (1) percentage of new residents who were assessed for pain with the PACSLAC-II on admission; (2) percentage of current residents assessed with the PACSLAC-II a minimum of once per week; (3) for residents with PACSLAC-II findings of moderate-to-severe pain, percentage of residents with a documented treatment plan within 24 hours of pain identification; (4) percentage of residents reassessed with a standardized pain assessment tool within 24 hours of treatment implementation; and (5) percentage of residents assessed for side effects within 24 hours of treatment implementation. Quality indicators were reported on a weekly basis by a pain champion from each LTC unit for the period of the study.

#### Demographic Questionnaire

Participants completed a demographic information sheet that included questions about their age, gender, years of experience working in LTC, and professional title (nurse or care aide).

#### Nursing Stress Scale

The Nursing Stress Scale (NSS) [28] is a 34-item scale comprising situations that could be perceived as stressful by frontline staff. Subscales include death and dying, conflict with supervisor, inadequate preparation, lack of support, conflict with coworker, workload, and uncertainty concerning treatment. Each item is rated on a 4-point Likert scale according to the frequency with which each item is felt to be stressful, that is, ranging from 0 (never) to 3 (very frequently). A higher total score indicates higher levels of perceived stress. For the purposes of this study, the original NSS was administered to nurses, and a modified version of the NSS was administered to care aides, that is, for items of the original NSS in which physicians were mentioned, the modified version of the NSS mentioned both physicians and nurses (eg, criticism by a physician in the original NSS was changed to criticism by a nurse or physician in the modified NSS). The NSS total score has demonstrated good test-retest reliability (ie, *r*=.81) and internal consistency (ie, Cronbach alpha=.89) [28]. In the same study, test-retest reliability was adequate for all subscales except for inadequate preparation (*r*=.42), lack of support (*r*=.65), and uncertainty concerning treatment (*r*=.68) [28]. Similarly, with regard to internal reliability, most subscales were adequately reliable, but conflict with supervisor, lack of support, and conflict with coworker had Cronbach alpha values of .68, .64, and .68 [28], respectively. In our study, internal consistency for total scores on the NSS was excellent (Cronbach alpha=.94). The death and dying, conflict with supervisor, inadequate preparation, lack of support, conflict with coworker, workload, and uncertainty concerning treatment subscales had Cronbach alpha values of .81, .67, .70, .73, .78, .83, and .79, respectively. All subscales, except for the conflict with supervisor subscale, had satisfactory reliability in our study. The reliability of the conflict with supervisor subscale was marginal.

#### Maslach Burnout Inventory

The Maslach Burnout Inventory (MBI) [29] is a 22-item scale comprising statements describing different experiences or situations encountered on the job for health care professionals. Subscales include emotional exhaustion, depersonalization, and personal accomplishment. Each item is rated on a 7-point Likert-type scale ranging from 0 (never) to 6 (every day) to assess the frequency of perceived burnout. A higher score is indicative of higher perceived burnout. With regard to test-retest reliability, correlations of each MBI subscales from the first and second administrations are between .53 and .69 [29]. The MBI has also demonstrated adequate internal consistency with a Cronbach alpha value of .84 for the total score and .86, .72, and .74, respectively, for emotional exhaustion, depersonalization, and personal accomplishment subscales [29]. In our study, internal consistency for total scores on the MBI was satisfactory (Cronbach alpha=.79). The emotional exhaustion, depersonalization, and personal accomplishment subscales had Cronbach alpha values of .89, .70, and .66, respectively. All subscales, except for the personal accomplishment subscale, had satisfactory reliability in our study. The internal consistency for personal accomplishment subscale was marginal.

#### Interviews

Semistructured interviews were conducted with the help of an interview guide designed specifically for this study (Textbox 1). The interview guide consisted of a series of questions regarding the attitudes toward pain assessment practices in older adults with dementia, barriers to effective pain management practices in LTC, and frontline staff experiences of using the tablet app version of the PACSLAC-II during the implementation period.

**Moderator guide for interviews with frontline staff.**

**Assessing pain among long-term care residents with dementia**

- What were some of the pain assessment tools that you had previously used to assess pain among long-term care residents?
- How would you describe your workload and stress levels when assessing pain in long-term care residents with dementia?
- What role did you play in assessing pain among residents with dementia in your facility?
- In what ways do you believe pain to be adequately or inadequately addressed for residents with dementia?

**Implementation of Pain Assessment Checklist for Seniors With Limited Ability to Communicate-II (PACSLAC-II)**

- Why would you or why would you not consider using a tablet app version of the PACSLAC-II?
- In what ways would it or would it not be feasible to use the tablet app version of the PACSLAC-II in your facility?
- How did the tablet app/paper/both versions of the PACSLAC-II affect your workload levels?
- How would you describe your experience using the tablet app version of the PACSLAC-II?
- Would you prefer the paper or the tablet app version of the PACSLAC-II to assess pain?

**Pain assessment practices**

- What are particular barriers or challenges to changing or improving pain assessment practices within your facility?
- What are the supporting factors that assist in changing or improving pain assessment practices within your facility?
- How are decisions about changes in pain assessment practices made within your facility?
- What role do you play in the decisions made about pain assessment practices?

### Procedure

Before the start of the study, a pain champion was appointed for each of the participating LTC units. This pain champion was responsible for overseeing the implementation of the standardized pain assessment protocol for the implementation period. This protocol comprised of the criteria assessed by the quality indicators (see *Quality Indicators* section in *Methods*). For this study, participants from each LTC unit took part in a baseline, training, implementation, and a follow-up period (Textbox 2).

**Description of the study’s methodology.**

**Baseline (3 weeks)**

- Quality indicators collected for each week.

**Training (9 weeks)**

- Web-based training program and/or in-person training.
- Training about the tablet app as needed.

**Implementation (6 weeks)**

- Assigned to use the tablet app, paper-and-pencil, or both methods of administration.
- Quality indicators collected for each week.
- Questionnaires completed at the end of the period.
- Interviews completed at the end of the period.

**Follow-up (3 weeks)**

- Quality indicators collected for each week.

#### Baseline Period

During the 3-week baseline period, participating LTC units collected the weekly quality indicators without making any changes to their pain assessment practices.

#### Training Period

Frontline staff in all participating LTC units were then trained on how to adequately assess pain using the PACSLAC-II and implement the standardized pain assessment protocol. A Web-based training program (see *Web-Based Training Program* section) in conjunction with in-person training was employed. The training was provided in both formats to ensure that we were able to provide the necessary information to the staff who was going to use the PACSLAC-II as part of this study. Our research team was able to provide direct training to 89 frontline staff members (including 18 members who completed the Web-based training program). Moreover, the pain champion at each facility was available as a resource for all staff who had questions or uncertainties about the assessment process and the pain protocol. For those LTC units randomized to use the tablet app (see *Implementation Period* section), in-person training on how to use different features of the tablet app was provided by a member of the research team to all frontline staff and printed instructions on how to use the tablet app were distributed to these LTC units (see *Tablet App Version of the Pain Assessment Checklist for Seniors With Limited Ability to Communicate-II* section).

#### Implementation Period

Following the training period, LTC units were randomized to use the paper-and-pencil, tablet app, or a combination of both methods of administration. Two tablets were used in the implementation period at each unit, and LTC units collected quality indicator data. The pain champion encouraged the use of the PACSLAC-II in accordance with the implementation protocol. The research team visited the LTC units on a regular basis to ensure that the implementation protocol was being adhered to throughout the implementation period. Tablets were made available to each unit during each of the tablet periods. During the 6-week implementation period, frontline staff completed the demographic information sheet as well as the MBI and NSS. Finally, following the implementation period, semistructured individual interviews were completed with interested frontline staff.

#### Follow-Up Period

During the 3-week follow-up period, participating LTC units collected the weekly quality indicators while continuing with the standardized pain assessment protocol. Regular visits and support from the research team did not take place during this period.

## Results

### Quality Indicators

For each quality indicator, percentages were averaged for both the baseline and implementation periods (Table 1). Consistent with a case series design [25], percentages were averaged separately for each participating LTC unit. Overall, as shown in Table 1, for units that had at least one admission during the implementation period, the average percentage of pain assessments with specialized assessment tools completed on admission was at 100% for all except one of the LTC units. The average percentage of current residents who underwent weekly pain assessments increased from the baseline to the implementation period. Improvements in treatment plan documentation and reassessment following the treatment plan implementation were found for all except 1 of the LTC units. Assessments of side effects following treatment plan implementation were not consistently observed across facilities. Furthermore, during the follow-up period, improvements in pain assessment practices identified during the implementation period were not maintained. Finally, an examination of the method of administration during the implementation period did not reveal apparent differences in averaged percentages for any of the quality indicators.

**Table 1:** Quality indicators from baseline, implementation, and follow-up periods averaged across the 3 weeks from each period.

Long-term care unit		QI^a^ 1^b^, % (n/N)	QI 2^c^, % (n/N)	QI 3^d^, % (n/N)	QI 4^e^, % (n/N)		QI 5^f^, % (n/N)	
**Unit A**								
	Baseline	—^g^	0 (0.00/31.67)^h^	73 (2.67/3.67)	9 (0.33/3.67)		0 (0.00/3.67)	
	Tablet app	—	100 (30.67/30.67)	100 (3.00/3.00)	100 (3.00/3.00)		0 (0.00/0.00)	
	Tablet app (continued)	100 (1.00/1.00)	100 (29.67/29.67)	100 (1.00/1.00)	100 (1.00/1.00)		0 (0.00/0.00)	
	Follow-up	0 (0.00/1.00)	2 (0.67/30.67)	100 (1.50/1.50)	100 (1.50/1.50)		67 (1.00/1.50)	
**Unit B**								
	Baseline	0 (0.00/1.50)	9 (2.33/25.67)	91 (3.33/3.67)	0 (0.00/3.67)		46 (1.67/3.67)	
	Paper-and-pencil	—	100 (18.00/18.00)	100 (1.00/1.00)	100 (1.00/1.00)		0 (0.00/1.00)	
	Tablet app	100 (1.00/1.00)	100 (19.33/19.33)	100 (1.00/1.00)	100 (1.00/1.00)		0 (0.00/1.00)	
	Follow-up	—	15 (3.33/22.00)	—	—		—	
**Unit C**								
	Baseline	—	0 (0.00/39.00)	100 (8.00/8.00)	4 (0.33/8.00)		8 (0.67/8.00)	
	Paper-and-pencil	—	100 (39.00/39.00)	100 (5.00/5.00)	100 (5.00/5.00)		0 (0.00/5.00)	
	Paper-and-pencil (continued)	—	100 (38.33/38.33)	100 (2.00/2.00)	100 (2.00/2.00)		0 (0.00/2.00)	
	Follow-up	—	0 (0.00/39.00)	100 (2.00/2.00)	100 (2.00/2.00)		0 (0.00/2.00)	
**Unit D**								
	Baseline	0 (0.00/1.00)	0 (0.00/38.33)	100 (1.50/1.50)	100 (1.50/1.50)		100 (1.50/1.50)	
	Paper-and-pencil	—	98 (38.33/39.00)	100 (1.00/1.00)	100 (1.00/1.00)		0 (0.00/1.00)	
	Tablet app	—	100 (38.33/38.33)	100 (1.50/1.50)	100 (1.50/1.50)		0 (0.00/1.50)	
	Follow-up	—	0 (0.00/39.00)	100 (1.00/1.00)	100 (1.00/1.00)		0 (0.00/1.00)	
**Unit E**								
	Baseline	0 (0.00/2.00)	5 (4.67/90.00)	93 (4.33/4.67)	14 (0.67/4.67)		0 (0.00/4.67)	
	Paper-and-pencil	0 (0.00/4.33)	65 (58.33/90.00)	100 (1.33/1.33)	50 (0.67/1.33)		100 (1.33/1.33)	
	Tablet app	0 (0.00/3.50)	10 (8.67/90.00)	—	—		—	
	Follow-up	0 (0.00/2.00)	8 (7.33/90.00)	—	—		—	
**Unit F**								
	Baseline	—	0 (0.00/30.00)	100 (2.00/2.00)	100 (2.00/2.00)		100 (2.00/2.00)	
	Tablet app	—	100 (30.00/30.00)	100 (4.00/4.00)	100 (4.00/4.00)		100 (4.00/4.00)	
	Paper-and-pencil	—	100 (30.00/30.00)	100 (4.33/4.33)	100 (4.33/4.33)		100 (4.33/4.33)	
	Follow-up	100 (1.00/1.00)	4 (1.33/30.00)	100 (1.67/1.67)	100 (1.67/1.67)		100 (1.67/1.67)	
**Unit G**								
	Baseline	100 (1.00/1.00)	0 (0.00/45.00)	84 (9.00/10.67)	0 (0.00/10.67)		69 (7.33/10.67)	
	Paper-and-pencil	—	51 (23.00/45.00)	8 (0.67/8.33)	4 (0.33/8.33)		0 (0.00/8.33)	
	Paper-and-pencil (continued)	100 (1.00/1.00)	29 (13.00/45.00)	0 (0.00/3.00)	0 (0.00/3.00)		0 (0.00/3.00)	
	Follow-up	—	16 (7.33/45.00)	30 (1.00/3.33)	10 (0.33/3.33)		10 (0.33/3.33)	

^a^QI: quality indicator.

^b^Number and percentage of new residents assessed using the PACSLAC-II on admission averaged across the total number of weeks from each period.

^c^Number and percentage of current residents assessed using the PACSLAC-II at least once per week averaged across the total number of weeks from each period.

^d^Number and percentage of residents with moderate-to-severe pain with a treatment plan within 24 hours averaged across the total number of weeks from each period.

^e^Number and percentage of residents with moderate-to-severe pain with a reassessment within 24 hours averaged across the total number of weeks from each period.

^f^Number and percentage of residents with moderate-to-severe pain assessed for side effects within 24 hours averaged across the total number of weeks from each period.

^g^The symbol "—" indicates that there were no new admissions or no residents with moderate-to-severe pain during for that period.

^h^The n and N values in this table have decimals as they represent total numbers collected on a weekly basis averaged over a period of 3 weeks.

### Questionnaires

Descriptive statistics and frequencies for demographic characteristics for each participating LTC unit are reported in Table 2.

The means and standard deviations for stress and burnout subscale scores for each condition are presented in Tables 3 and 4, respectively. Before conducting our principal analyses, we examined the relationship between the demographic characteristics of experience, age, gender, and professional title (ie, nurses or care aide) and subscale scores on stress and burnout measures using Pearson correlations. Of all the correlations, age was significantly related to conflict with physicians subscale (*r*=−.22, *P*=.04), and job title was significantly related to death and dying (*r*=.25, *P*=.02), conflict with physicians (*r<*.29, *P*=.006), and workload (*r*=.28, *P*=.008) subscales, with nurses scoring higher than care aides. Therefore, these demographic characteristics were included as covariates in subsequent analyses involving the corresponding subscale scores. To test the significance of mean differences among conditions, a series of analyses of variance (ANOVA) and covariance (ANCOVA) with conditions (ie, *paper-and-pencil only*, *paper-and-pencil followed by tablet app*, *tablet app followed by paper-and-pencil*, and *tablet app only*) as the between-subjects factor were conducted. The first series of ANOVAs and ANCOVAs examined stress subscale scores (ie, death and dying, conflict with physicians, inadequate preparation, lack of staff support, conflict with other nurses, workload, and uncertainty concerning treatment). The second series of ANOVAs examined burnout subscale scores (ie, emotional exhaustion, depersonalization, and personal achievement) at the end of the implementation period. It was expected that stress and burnout levels would be lower in conditions in which frontline staff had most recently used the tablet app method of administration rather than the paper-and-pencil method of administration.

With regard to stress subscale scores, the ANCOVA for workload (with nurse vs care aide as covariates) revealed a significant between-subjects effect (*F*_3,86_=8.29, *P*<.001, partial *η^2^*=0.22). Follow-up pairwise comparisons showed that frontline staff in the *paper-and-pencil followed by tablet app* condition reported significantly lower levels of workload compared with the *paper-and-pencil only* (*P*<.001) and *tablet app followed by paper-and-pencil* (*P*<.001) conditions. Frontline staff also reported lower levels of workload in the *tablet only* condition compared with the *paper-and-pencil only* condition (*P*=.05). No other differences for workload were found. The ANOVA and ANCOVA analyses for death and dying, conflict with physicians, inadequate preparation, lack of staff support, conflict with other nurses, and uncertainty concerning treatment did not show any significant effects.

With regard to burnout subscale scores, the ANOVA for emotional exhaustion revealed a significant between-subjects effect (*F*_3,88_=4.86, *P*=.004, partial *η^2^*=0.14). Follow-up pairwise comparisons showed that frontline staff in the *paper-and-pencil followed by tablet app* condition reported significantly lower levels of emotional exhaustion compared with the *paper-and-pencil only* (*P*=.002) and *tablet app followed by paper-and-pencil* (*P*=.002) conditions. No other significant differences in emotional exhaustion were found. The ANOVA for depersonalization revealed a significant between-subjects effect (*F*_3,88_=9.54, *P*<.001, partial *η^2^*=0.25). Follow-up pairwise comparisons showed that frontline staff in the *paper-and-pencil only* condition reported significantly higher levels of depersonalization compared with the *tablet app only* (*P*=.008), *paper-and-pencil followed by tablet app* (*P*<.001), and *tablet app followed by paper-and-pencil* (*P*<.001) conditions. No other significant differences in depersonalization were found. The ANOVA for personal achievement did not show any significant effects.

**Table 2:** Demographic characteristics for each participating long-term care unit.

Long-term care unit	Participants, N	Experience (years), mean (SD)	Age (years), mean (SD)	Gender (female), n (%)	Title (nurses), n (%)
Unit A	16	10.25 (6.73)	44.81 (11.35)	12 (75)	3 (19)
Unit B	21	10.52 (8.00)	40.70 (9.68)	18 (86)	6 (29)
Unit C	16	10.38 (7.86)	39.60 (9.60)	16 (100)	5 (31)
Unit D	20	8.35 (7.96)	41.26 (10.07)	18 (90)	8 (40)
Unit E	24	9.08 (8.45)	44.08 (12.07)	23 (96)	7 (29)
Unit F	17	13.65 (10.53)	43.31 (10.06)	17 (100)	3 (18)
Unit G	7	11.43 (11.47)	39.71 (16.01)	7 (100)	1 (14)

**Table 3:** Stress subscale scores for each condition.

Condition	Count, n	Stress subscale scores, mean (SD)						
		Death and dying	Conflict with supervisor	Inadequate preparation	Lack of support	Conflict with coworker	Workload	Uncertainty concerning treatment
Paper-and-pencil only	18	7.44 (3.57)	4.53 (2.65)	3.39 (2.00)	1.72 (2.05)	4.50 (3.15)	8.82 (4.45)	4.11 (2.78)
Tablet app only	12	8.33 (2.96)	4.42 (2.58)	3.08 (0.79)	2.25 (1.49)	4.08 (1.83)	6.17 (2.52)	4.33 (2.46)
Paper-and-pencil followed by tablet app	31	6.55 (2.59)	3.21 (1.66)	2.42 (1.06)	1.84 (1.32)	2.97 (2.17)	4.87 (2.59)	2.87 (2.16)
Tablet app followed by paper-and-pencil	31	6.87 (3.67)	3.60 (2.54)	2.65 (1.38)	2.48 (1.96)	4.45 (3.29)	8.48 (4.05)	4.26 (2.80)

**Table 4:** Burnout subscale scores for each condition.

Condition	Count, n	Burnout subscale scores, mean (SD)		
		Emotional exhaustion	Depersonalization	Personal accomplishment
Paper-and-pencil only	18	24.22 (11.47)	9.50 (6.51)	36.44 (7.32)
Tablet app only	12	18.50 (9.41)	5.17 (4.65)	35.92 (6.50)
Paper-and-pencil followed by tablet app	31	14.48 (7.82)	2.94 (3.12)	39.07 (5.09)
Tablet app followed by paper-and-pencil	31	23.23 (12.49)	4.10 (3.43)	38.81 (7.00)

### Interviews

We interviewed a total of 43 participants. Saturation was achieved, given that we observed that the addition of the last several interviews was not yielding new information. Responses from interviews were analyzed using thematic content analysis [30]. By identifying common themes in the data set, researchers can capture and make sense of the meanings and experiences of participants in relation to the questions being asked. In accordance with the outline provided by Braun and Clarke [30], the primary researcher began the data analysis by transcribing all the interviews. Because a deductive approach was taken, the themes were constructed around the 3 main content areas of the interview guide (ie, user-friendliness, feasibility, and overall impression of the PACSLAC-II). Slight variations in main content areas across the LTC units, however, were observed. That is, LTC units randomized to use both versions of the PACSLAC-II were asked about all of the main content areas, LTC units randomized to use the tablet app version of the PACSLAC-II were asked about all of the main content areas with a focus on user-friendliness, and LTC units randomized to use the paper-and-pencil version of the PACSLAC-II were asked about all of the main content areas except for user-friendliness. Following the identification of the main content areas, the researcher completed constant reading, rereading, and highlighting of important and reoccurring aspects of the transcripts to become more familiar with the textual data for proper coding. Codes (ie, units of text that represents a meaningful idea relating to the identified content areas) were identified using NVivo (QSR International, Melbourne, Australia). The identified codes from the text were then reviewed and organized into themes. A second coder then organized the narrative responses of 21% (9/43) of the participants into these thematic categories to ensure consistency of the coding and trustworthiness of themes. Disagreements were resolved by consensus or if consensus could not be reached, reorganization of thematic categories. Representative quotes from each theme are shown in Textbox 3.

**Quotes representative of each identified theme.**

**User-friendliness**

Benefits of the visual features

- “It was nice because you can go back and see the ups and downs from each of the graphs. It was nice, it was visual. Whereas paper, it’s 10/10 pain or 7/10 pain and it’s like where are you at. But with the graph, you could see, it’s visual.”
- “I think a visual graph when you can see it, and you see the peak times, and from there you can implement probably a medication that would work better or start something and see.”

Faster and easier access of data

- “Yes, with tablet I think once it’s filled out it’s collated, cause with paper form, I have to do the photocopy and make sure that we have another copy in the chart and then the follow-up, right, and within 24 hours, you have to do another PACSLAC if they are in pain.”
- “When using paper, you have to write, and it would take time… But for the tablet, all you have to do is tap. It’s much faster… It’s time-wise when using tablet.”

Preference for the tablet app version of the Pain Assessment Checklist for Seniors With Limited Ability to Communicate-II (PACSLAC-II)

- “Myself, tablet version because, like I said, we can go back and see the graphs and how they are responding and not responding, where the pain lies, do they need something extra. Paper version, definitely if something like that goes out, we have the option of using the paper always, that’s always nice to have to keep track. Just for me, visually, I like using the tablet way better.”
- “For me, it would be better if we had the tablet version. Firstly, it saves money. Imagine printing. But if you have it on tablet, you can just click, click. Storing is better than having the paper. It would appear cheaper in the long run.”

**Feasibility**

Barriers associated with the tablet app version of the PACSLAC-II

- “Sometimes the tablet is not working because of the connection but it’s easier to use the tablet…”
- “I think the tablet version is a great idea. I think that if you were going to implement it, it would just be getting its own Wi-Fi connection because obviously we struggle with the Wi-Fi connection.”

Barriers associated with changes in pain assessment practices

- “Like I said, we look after these people, we’re here more often with these people than we are with our own families. So, we know these people inside and out and so when we say that there’s an issue or this person’s off or they look like they’re having a lot more pain, trust us….the doctor’s only here once a week and he spends not very much time with these people and he comes in and he does his two minute assessment and says, ‘they look fine today, no let’s hold off.’ Really, now we have to go another seven whole days of more documentation for him to say, ‘well, I really don’t know, we’ll bump them up a little bit.’ So, you know what I’m saying, it’s the frustration of not being heard.”
- “I think we should try to get everyone on the same page for care and management because it just seems that some nurses will do certain things and others won’t.”

**Overall impression of the PACSLAC-II**

No significant increase in workload levels with implementation of PACSLAC-II

- “It means more paperwork, but it’s the paperwork that will help us in the end, and it doesn't take too long, maybe three or four minutes.”
- “It honestly didn’t take them that long. It was very quick, and I don’t think it was that time-consuming.”

Overall positive impression of the PACSLAC-II

- “I guess, overall experience using the PACSLAC, I think it was a win-win.”
- “I think it kind of gets everybody on the same page, you know, we can discuss it afterwards and the continuing care aides would talk about it too and it kind of makes you think.”

#### User-Friendliness

As shown in Textbox 3, 3 themes emerged pertaining to the user-friendliness of the tablet app version of the PACSLAC-II. The first theme focused on the benefits of the visual features of the tablet app version. That is, frontline staff spoke of the value of being able to identify visual patterns in pain scores over time using a graph instead of numbers. They noted that this feature was helpful in terms of pain assessment and pain management. The second theme related to faster and easier access to data; the consensus among the frontline staff was that the paper-and-pencil version of the PACSLAC-II resulted in extra paperwork that affected routine pain assessment practices. In contrast, the tablet app version provided a faster and easier way to store and access data. The third theme centered around a preference for the tablet app version of the PACSLAC-II. Most frontline staff preferred the tablet app version, although some of the frontline staff noted hesitations about the tablet app version related to connectivity issues.

#### Feasibility

Regarding feasibility, 2 themes were recognized (Textbox 3). The first theme identified related to barriers associated with the tablet app version of the PACSLAC-II and the second theme related to barriers associated with changes in pain assessment practices. The primary barrier associated with the use of the tablet app version was connectivity. The tablet was connected to a wireless internet connection that was, occasionally, unreliable. Thus, significant challenges in consistently using the tablet app version when needed were noted.

Given that pain assessments were only required a minimum of once a week, we were advised that staff completed the pain assessments the following day if the technology did not work as planned. In the very small number of instances that there was a pressing need for an immediate pain assessment and the app was not working (eg, because of a connectivity problem), the PACSLAC-II was completed on paper, and the information was entered into the app the following day.

The primary barrier associated with changes in pain assessment practices identified by frontline staff was miscommunication across disciplines (ie, physicians, nurses, and care aides). Specifically, care aides noted that they sometimes felt dismissed when they would report to a nurse that one of the LTC residents was in pain. Another challenge was having physicians agree with nurses on the need for pain relief for LTC residents.

#### Overall Impression of the Pain Assessment Checklist for Seniors With Limited Ability to Communicate-II

The overall impression of frontline staff of the implementation period was another main area of focus for the interviews, and 2 main themes emerged that pertained to the overall impression of the frontline staff (Textbox 3). With regard to the first theme, the majority of frontline staff reported that they did not notice a significant increase in workload levels and that, even if an increase in workload were to occur, that it would be worthwhile. However, some frontline staff—most notably the nurse manager or pain champion—indicated additional workload stemming from paperwork and management of the implementation because of their unit’s participation in the research aspects of the study and the tracking of the quality indicators. With regard to the second theme, the majority of frontline staff reported that their experience with using the PACSLAC-II during the implementation period was positive. Of note, some participants noted that the use of the PACSLAC-II provided a common language for staff across disciplines to talk about pain.

## Discussion

### Principal Findings

The implementation of the PACSLAC-II in this study resulted in improvements in pain assessment frequency regardless of the medium used for the PACSLAC-II. Importantly, the percentage of new residents assessed on admission and current residents assessed on a weekly basis increased to 100% for all except 1 participating LTC unit regardless of the medium of administration that was employed. The tablet app version of the PACSLAC-II was also associated with lower levels of reported stress and burnout among staff. For example, frontline staff who were in the *paper-and-pencil only* and *tablet app to paper-and-pencil* conditions experienced significantly higher levels of emotional exhaustion and workload compared with frontline staff who were in the *paper-and-pencil to tablet app* condition. Perhaps the benefit of a reduced workload because of the tablet (when it was preceded by paper and pencil administrations) became more evident and was better appreciated by those who saw the change by experiencing the paper-and-pencil version first. Similarly, those who started with the tablet version would have reported a greater workload following the paper and pencil version because of the contrast between the two conditions. Unsurprisingly, frontline staff in the *tablet app only* condition also reported lower levels of workload compared with their counterparts in the *paper-and-pencil only* condition. Furthermore, those in the *paper-and-pencil only* condition experienced significantly higher levels of depersonalization than all other conditions.

Finally, frontline staff responses during interviews suggested an overall preference for the tablet app version. This overall preference is a very important theme because, through the identification of preferences, we can recognize the features of the tablet app that prove to be beneficial in improving pain assessment practices. It also shows potential for the PACSLAC-II to be utilized on a more regular basis if the tablet app version is employed by LTC facilities. We are hoping to incorporate additional features in a future version of the app that could automatize the tracking of quality indicators (eg, percentage of residents assessed at least once a week), facilitating the monitoring of quality improvement programs without extra burden for staff. Another possible feature would be to incorporate the capability for generating reminders for staff about which residents are due for an assessment.

Based on the qualitative analysis, we were very encouraged by staff who observed that the PACSLAC-II gave them a common language to discuss pain across disciplines. Diarized PACSLAC-II scores could also assist physicians who visit the facility intermittently and who would otherwise not have an ongoing index of the patients’ day-to-day pain.

### Limitations and Future Directions

Improvements in pain assessment frequency were not maintained during the follow-up period. Improvement in aspects of treatment follow-up for residents with moderate-to-severe pain (ie, treatment plan implemented within 24 hours, reassessed for pain within 24 hours of the treatment plan, and assessment for side effects of treatment) was also not consistently found across LTC facilities. Also, differences in pain assessment frequency and treatment follow-up as a function of the tablet app and paper-and-pencil versions of the PACSLAC-II were not identified. Furthermore, although an overall preference for the tablet app was identified, one issue that would need to be resolved to increase the uptake of this method of administration is improved connectivity.

Several directions regarding the standardized pain assessment protocol should be considered for future research. This study was carried out over a 12-week period. Future research should focus on the study of the different modes of administration of the PACSLAC-II over the longer term. To ensure the feasibility of integrating the use of the tablet app version in LTC facilities, future research could aim for longer implementation and follow-up periods, especially given the trend of decreased pain assessment frequency and follow-up treatment during the follow-up periods in this study. Furthermore, many of the residents who experienced moderate-to-severe pain did not have documentation in their charts to indicate whether or not they were checked for side effects 24 hours following treatment. Moreover, in this study, we measured whether a treatment plan was implemented when moderate-to-severe pain was identified, but we did not measure the types of treatment plans that were implemented (eg, medications or repositioning). Research into these aspects of treatment follow-up should be explored in future studies. 

Other avenues for future research may include the addition and study of new PACSLAC-II app features. We plan to use lessons learned from this study as we develop the next version of our app. Such features could include automatic detection of significant changes in residents’ regular pattern of scores, access to educational resources and tools, and ability to track average facility-wide pain levels. This latter feature could allow for the use of facility-wide PACSLAC-II scores as a quality indicator with respect to progress in managing pain. Moreover, although the tablet app method of administration was meant to be a simple and literal implementation of the paper-and-pencil method of administration, the inclusion of a user experience/interface design could have nevertheless resulted in a tablet app version of the PACSLAC-II that was better designed for the user.

In this study, many of our participants were trained in pain assessment and use of the PACSLAC-II through a Web-based training program that we had previously validated using a paper and pencil version of the app. We are planning to integrate specific app training in a future version of our training program and then study the contribution of the app-specific component on app use and benefits. Another area for future research would be the development of a protocol for steps to take when technical issues arise.

### Conclusions

Given that observational pain assessment tools are not utilized as frequently in LTC facilities as they should be, it is important to introduce innovative ways in which pain assessment practices could be improved. One such innovation is the use of technology to facilitate regular pain assessments. Findings from this study support the use of either version of the PACSLAC-II when implementing a standardized pain assessment protocol as both versions were found to improve pain assessment frequency and some aspects of treatment follow-up. However, the tablet app version of the PACSLAC-II was preferred by frontline staff and seemed to have resulted in lower levels of reported emotional exhaustion, depersonalization, and workload. More research regarding the use of technological innovations for pain assessments in LTC facilities, therefore, has the potential to lead to improved pain assessments, and an associated increase in quality of life, for LTC residents.

## Abbreviations

ANCOVA: analysis of covariance
ANOVA: analysis of variance
LTC: long-term care
MBI: Maslach Burnout Inventory
mHealth: mobile health
NSS: Nursing Stress Scale
PACSLAC: Pain Assessment Checklist for Seniors With Limited Ability to Communicate
